# NRF3-POMP-20S Proteasome Assembly Axis Promotes Cancer Development via Ubiquitin-Independent Proteolysis of p53 and Retinoblastoma Protein

**DOI:** 10.1128/MCB.00597-19

**Published:** 2020-04-28

**Authors:** Tsuyoshi Waku, Nanami Nakamura, Misaki Koji, Hidenori Watanabe, Hiroki Katoh, Chika Tatsumi, Natsuko Tamura, Atsushi Hatanaka, Shuuhei Hirose, Hiroyuki Katayama, Misato Tani, Yuki Kubo, Jun Hamazaki, Takao Hamakubo, Akira Watanabe, Shigeo Murata, Akira Kobayashi

**Affiliations:** a Laboratory for Genetic Code, Department of Medical Life Systems, Faculty of Life and Medical Sciences, Doshisha University, Kyoto, Japan; b Laboratory for Genetic Code, Graduate School of Life and Medical Sciences, Doshisha University, Kyoto, Japan; c Japan Society for the Promotion of Science, Tokyo, Japan; d Laboratory of Protein Metabolism, Graduate School of Pharmaceutical Sciences, The University of Tokyo, Tokyo, Japan; e Department of Protein-Protein Interaction Research, Institute for Advanced Medical Sciences, Nippon Medical School, Kanagawa, Japan; f Department of Life Science Frontiers, Center for iPS Cell Research and Application, Kyoto University, Kyoto, Japan

**Keywords:** 20S proteasome assembly, cancer development, colorectal cancer, NRF3, NFE2L3, POMP, Rb, retinoblastoma, ubiquitin-independent proteolysis, p53, TP53

## Abstract

Proteasomes are essential protease complexes that maintain cellular homeostasis, and aberrant proteasomal activity supports cancer development. The regulatory mechanisms and biological function of the ubiquitin-26S proteasome have been studied extensively, while those of the ubiquitin-independent 20S proteasome system remain obscure. Here, we show that the cap ’n’ collar (CNC) family transcription factor NRF3 specifically enhances 20S proteasome assembly in cancer cells and that 20S proteasomes contribute to colorectal cancer development through ubiquitin-independent proteolysis of the tumor suppressor p53 and retinoblastoma (Rb) proteins. The *NRF3* gene is highly expressed in many cancer tissues and cell lines and is important for cancer cell growth. In cancer cells, NRF3 upregulates the assembly of the 20S proteasome by directly inducing the gene expression of the 20S proteasome maturation protein POMP. Interestingly, NRF3 knockdown not only increases p53 and Rb protein levels but also increases p53 activities for tumor suppression, including cell cycle arrest and induction of apoptosis. Furthermore, protein stability and cell viability assays using two distinct proteasome inhibitor anticancer drugs, the 20S proteasome inhibitor bortezomib and the ubiquitin-activating enzyme E1 inhibitor TAK-243, show that the upregulation of the NRF3-POMP axis leads to ubiquitin-independent proteolysis of p53 and Rb and to impaired sensitivity to bortezomib but not TAK-243. More importantly, the NRF3-POMP axis supports tumorigenesis and metastasis, with higher *NRF3/POMP* expression levels correlating with poor prognoses in patients with colorectal or rectal adenocarcinoma. These results suggest that the NRF3-POMP-20S proteasome assembly axis is significant for cancer development via ubiquitin-independent proteolysis of tumor suppressor proteins.

## Abstract (precis)

Proteasomes are essential protease complexes that maintain cellular homeostasis, and aberrant proteasomal activity supports cancer development. The regulatory mechanisms and biological function of the ubiquitin-26S proteasome have been studied extensively, while those of the ubiquitin-independent 20S proteasome system remain obscure. Here, we show that the cap ’n’ collar (CNC) family transcription factor NRF3 specifically enhances 20S proteasome assembly in cancer cells and that 20S proteasomes contribute to colorectal cancer development through ubiquitin-independent proteolysis of the tumor suppressor p53 and retinoblastoma (Rb) proteins.

## INTRODUCTION

Cells acquire numerous hallmarks during cancer development, including sustained proliferation and resistance to apoptosis, which activate invasion and metastasis (1). Many of these phenotypic traits are brought about directly by genetic mutation, and several cancer phenotypes are further accelerated by aberrant proteolytic activity in a genetic-mutation-independent manner (2). The proteasome is a protease complex that is essential for maintaining cellular protein homeostasis. It consists of a 20S proteasome for protein degradation and a 19S regulatory particle (19S-RP) for ubiquitin recognition (3). The 26S proteasome, which is made up of a 20S proteasome and one or two 19S-RPs, regulates ubiquitin-dependent protein degradation, and the ubiquitin-26S proteasome system (UPS) has been implicated in the inactivation of the tumor suppressor p53 (TP53). As such, the role of the UPS in cancer has been researched extensively. Meanwhile, the 20S proteasome alone has been observed in both normal and cancer cells. Interestingly, the 20S proteasome physically interacts with several tumor suppressors, including p53 and retinoblastoma protein (Rb) (4, 5). However, the regulatory mechanisms and biological roles of the ubiquitin-independent 20S proteasome system in cancer cells remain obscure.

NRF3 (nuclear factor erythroid-2-like 3 [NFE2L3]) belongs to the cap ’n’ collar (CNC) family of transcription factors along with NRF1 (nuclear factor erythroid-2-like 1 [NFE2L1]) and NRF2 (nuclear factor erythroid-2-like 2 [NFE2L2]). The NRF3 and NRF1 proteins are segregated at the endoplasmic reticulum (ER). Upon exposure to stress and/or a signal, both NRF proteins are processed by the aspartic protease DDI2 (DNA damage inducible 1 homolog 2), inducing their nuclear translocation and transcriptional activation (6, 7). NRF1 induces the expression of almost all proteasome-related genes upon proteasome inhibition (8–10), although it has been unknown whether NRF3 is linked to proteasome regulation. Meanwhile, *NRF3* is among the 127 significantly mutated genes (SMGs) together with a well-known cancer-driving gene, *NRF2* (11). Cancer-associated NRF2 mutation hot spots in the N-terminal region, which are crucial for the interaction with the redox sensor KEAP1, result in a gain of function and cancer development (see Fig. S1A, top, in the supplemental material) (12). However, there are no hot spots in the NRF3 gene body and no mutations in its N-terminal region, which contains an ER anchor sequence and a DDI2-processing site (Fig. S1A, bottom), implying that these NRF3 mutations are passenger mutations that hardly affect molecular function. These insights suggest that NRF3 regulates the proteasome in cancer cells, although this remains unclear.

Here, we show that NRF3 upregulates the assembly of the 20S proteasome by directly inducing the expression of the gene encoding the 20S proteasome assembly chaperone, *POMP* (proteasome maturation protein). The NRF3-POMP axis further contributes to the ubiquitin-independent proteolysis of p53 and Rb and resistance to the proteasome inhibitor anticancer drug bortezomib (BTZ). More importantly, upregulation of the axis promotes tumor growth and metastasis *in vivo*, and colorectal cancer patients with higher *POMP/NRF3* expression levels exhibit lower overall and disease-free survival rates.

## RESULTS

### NRF3 positively regulates cancer cell growth and 20S proteasome activity.

**FIG 1:**
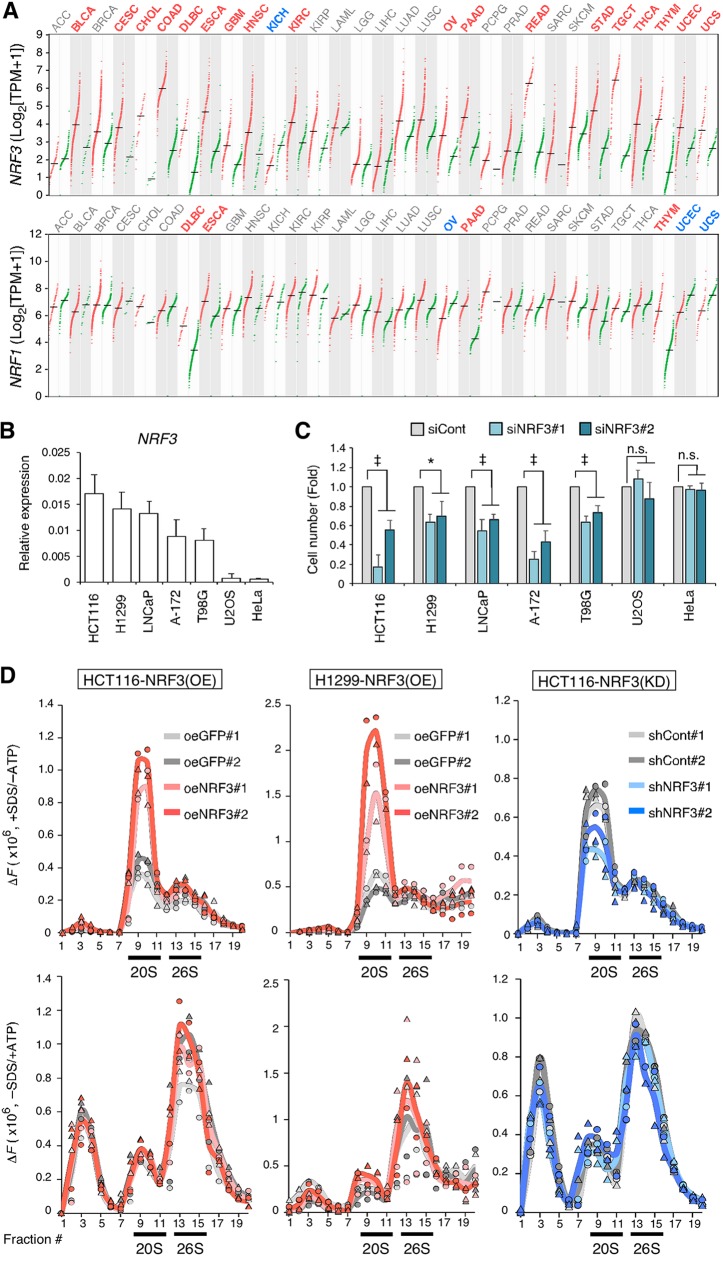
NRF3 sustains cancer cell growth and enhances 20S proteasome activity. (A) Dot plots showing *NRF3* (top) and *NRF1* (bottom) gene expression levels across multiple cancer types and paired normal samples. Red and green dots represent RNA sequencing expression values of patient-matched tumors and adjacent normal tissue archived at TCGA and the GTEx database. Red and blue abbreviations at the top of each graph indicate cancer types with significantly high and low expression levels of each *NRF* gene compared to normal samples, respectively (TPM, transcripts per million) (*q* value cutoff of 0.01 by ANOVA). The numbers of specimens are summarized in Table S3 in the supplemental material. ACC, adrenocortical carcinoma; BLCA, bladder urothelial carcinoma; BRCA, breast invasive carcinoma; CESC, cervical squamous cell carcinoma and endocervical adenocarcinoma; CHOL, cholangial carcinoma; COAD, colon adenocarcinoma; DLBC, lymphoid neoplasm diffuse large B-cell lymphoma; ESCA, esophageal carcinoma; GBM, glioblastoma multiforme; HNSC, head and neck squamous cell carcinoma; KICH, kidney chromophobe; KIRC, kidney renal clear cell carcinoma; KIRP, kidney renal papillary cell carcinoma; LAML, acute myeloid leukemia; LGG, brain lower-grade glioma; LIHC, liver hepatocellular carcinoma; LUAD, lung adenocarcinoma; LUSC, lung squamous cell carcinoma; OV, ovarian serous cystadenocarcinoma; PAAD, pancreatic adenocarcinoma; PCPG, pheochromocytoma and paraganglioma; PRAD, prostate adenocarcinoma; READ, rectal adenocarcinoma; SARC, sarcoma; SKCM, skin cutaneous melanoma; STAD, stomach adenocarcinoma; TGCT, testicular germ cell tumor; THCA, thyroid carcinoma; THYM, thymoma; UCEC, uterine corpus endometrial carcinoma; UCS, uterine carcinosarcoma. (B) Endogenous *NRF3* mRNA levels in HCT116 (colorectal carcinoma), H1299 (non-small-cell lung cancer), LNCaP (prostate adenocarcinoma), A-172 (glioblastoma), T98G (glioblastoma multiforme), U2OS (bone osteosarcoma), and HeLa (cervical adenocarcinoma) cell lines. *NRF3* mRNA levels were assessed by RT-qPCR (*n* = 3; means + standard deviations [SD]). (C) Impact of NRF3 knockdown on cell viability. NRF3 or control siRNA (siCont) was transfected into the indicated cells. After 48 h, the cells were counted using a hemocytometer. *, *P* < 0.05; ‡, *P* < 0.005; n.s., not significant (*n* = 3; means + SD) (determined by ANOVA followed by a Tukey test). (D) Impact of NRF3 overexpression [NRF3(OE)] or knockdown [NRF3(KD)] on proteasome activity. The indicated cell extracts were fractionated into 20 fractions using 10% to 40% glycerol gradient centrifugation and assayed for succinyl-Leu-Leu-Val-Tyr-7-amino-4-methylcoumarin (Suc-LLVY-AMC; chymotrypsin-like)-hydrolyzing activity of 20S proteasomes (+SDS/−ATP) (top) or 26S proteasomes (−SDS/+ATP) (bottom). The mean and individual values are represented as lines and marks, respectively (*n* = 2). The activity in fractions 1 to 5 was derived from nonproteasomal proteases. The NRF3 overexpression and shNRF3 stable-expression cell lines were represented as oeNRF3 and shNRF3, respectively. GFP overexpression (oeGFP) and control shRNA stable-expression (shCont) cell lines were used as controls.

First, we compared the expression levels of *NRF3* and *NRF1* in various cancer tissues and found that *NRF3* mRNA was more abundant in far more tumor specimens than normal specimens, particularly in colorectal adenocarcinoma (COAD), rectal adenocarcinoma (READ), and testicular germ cell tumors (TGCTs) (Fig. 1A, top). In contrast, *NRF1* mRNA levels were equally abundant between almost all tumor and normal specimens (Fig. 1A, bottom). These data suggest an association between cancer development and NRF3 but not NRF1. In addition, we report high NRF3 expression levels in the HCT116 (colorectal carcinoma), H1299 (non-small-cell lung cancer), LNCaP (prostate adenocarcinoma), A-172 (glioblastoma), and T98G (glioblastoma multiforme) cell lines but not in the U2OS (bone osteosarcoma) and HeLa (cervical adenocarcinoma) cell lines (Fig. 1B; Fig. S2A). Multiple immunoblot bands of NRF3 proteins indicate distinct forms with DDI2-mediated protein processing and/or other posttranslational modifications such as phosphorylation and ubiquitination (Fig. S2A). NRF3 knockdown significantly inhibited the growth of cancer cells with high expression levels of endogenous *NRF3* (Fig. 1C).

To investigate the impact of *NRF3* expression on proteasome activity, we generated NRF3 overexpression or knockdown HCT116 and H1299 cell lines (Fig. S2B) and confirmed that NRF3 proteins were abundant in the nuclear fraction of the overexpression cells (Fig. S2C). Next, we performed well-established proteasome activity assays using these cell lines (see Materials and Methods for details). Briefly, 20S and 26S proteasomes in cell lysates were fractionated by glycerol gradient centrifugation. Their activities were measured using a fluorogenic substrate with sodium dodecyl sulfate (SDS) or ATP, required for the *in vitro* detection of 20S or 26S proteasome activity, respectively (13). Comparison of these two conditions, the presence of SDS and the absence of ATP (+SDS/−ATP) and the absence of SDS and the presence of ATP (−SDS/+ATP), indicated that the peak in fractions 9 to 11 increased in an SDS-dependent manner, while the peak in fractions 13 to 15 increased in an ATP-dependent manner (Fig. S2D). Furthermore, both peaks were completely abolished by the proteasome inhibitor MG-132 (Fig. S2E, gray versus blue), indicating that the peaks in fractions 9 to 11 and 13 to 15 represent 20S and 26S proteasome activities, respectively. Meanwhile, a protease inhibitor cocktail (PIC) decreased the peak in fractions 1 to 5 (Fig. S2E, gray versus red), indicating nonproteasomal protease activity. In this assay, we revealed that NRF3 overexpression increased 20S proteasome activity in fractions 9 to 11 (Fig. 1D, top left and top middle; Fig. S2F). However, NRF3 overexpression barely influenced 26S proteasome activity in fractions 13 to 15 (Fig. 1D, bottom left and bottom middle), suggesting that NRF3 is a potent regulator of the 20S proteasome in cancer cells. Results obtained from NRF3 knockdown cells were consistent with these findings (Fig. 1D, top right and bottom right), although the effect of NRF3 knockdown on 20S proteasome activity was weaker than the effect of NRF3 overexpression. The difference between NRF3 overexpression and knockdown is addressed below (see Fig. 4; Fig. S3).

### NRF3 increases mRNA levels of *POMP*, which encodes a chaperone involved in 20S proteasome assembly.

**FIG 2:**
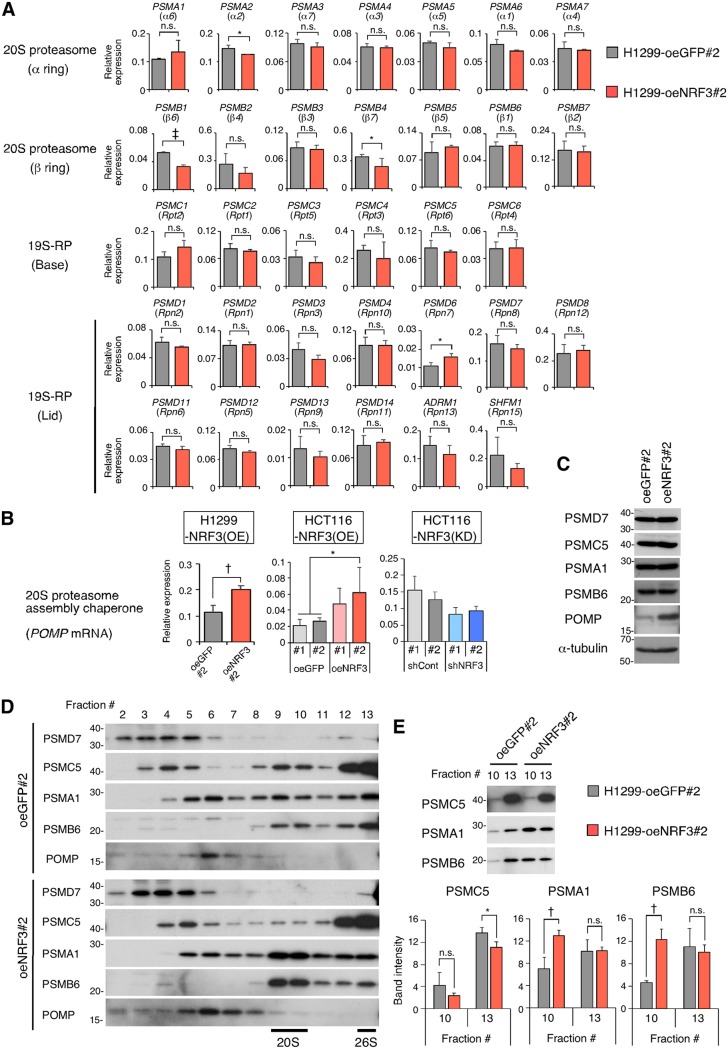
NRF3 overexpression induces *POMP* gene expression and 20S proteasome assembly. (A and B) Impact of NRF3 overexpression or knockdown on mRNA levels of 33 proteasome subunits (A) or a 20S proteasome assembly chaperone, *POMP* (B). The indicated mRNA levels in the H1299-NRF3(OE), HCT16-NRF3(OE), or HCT116-NRF3(KD) cell line were assessed by RT-qPCR. *, *P* < 0.05; †, *P* < 0.01; ‡, *P* < 0.005; n.s., not significant (*n* = 3; means + SD) (determined by *t* tests). (C) Impact of NRF3 overexpression on protein levels of proteasome subunits or a 20S proteasome assembly chaperone. The indicated proteins in H1299-oeNRF3#2 or -oeGFP#2 cells were detected by immunoblotting. (D and E) Impact of NRF3 overexpression on proteasome assembly. Fractions of H1299-oeNRF3#2 or -oeGFP#2 cells in Fig. 1D were immunoblotted for the indicated proteins in distinct SDS-PAGE gels (D) or in a single SDS-PAGE gel (E). In panel E, the expression values of the indicated proteins in H1299-oeNRF3#2 or -oeGFP#2 cells were assessed by immunoblotting and are presented in bar graphs. *, *P* < 0.05; †, *P* < 0.01; n.s., not significant (*n* = 3; means + SD) (determined by *t* tests).

**FIG 3:**
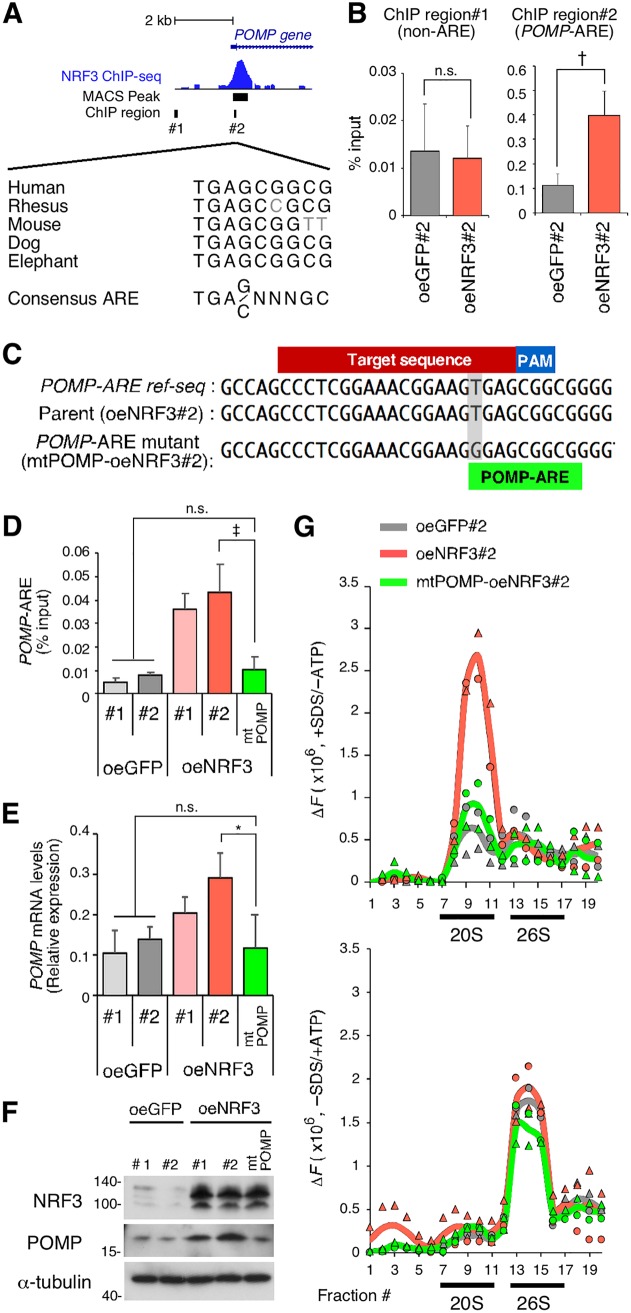
NRF3 overexpression enhances 20S proteasome activity by directly inducing *POMP* gene expression. (A) Genomic locus of the *POMP* gene with an NRF3 ChIP sequencing peak. Multiple sequences of a candidate ARE within the *POMP* promoter are indicated for different species. (B) ChIP-qPCR validation of NRF3 recruitment to the *POMP* promoter. H1299-oeNRF3#2 or -oeGFP#2 cells were subjected to ChIP assays using anti-NRF3 antibodies. Immunoprecipitated DNA was assessed by RT-qPCR assays using primers specific for each genomic region, as indicated in panel A. †, *P* < 0.01; n.s., not significant (*n* = 3; means ± SD) (determined by a *t* test). (C) CRISPR/Cas9-based mutagenesis of the ARE within the *POMP* promoter. *POMP*-ARE mutant cells (mtPOMP) were generated from H1299-oeNRF3#2 cells (parental). The protospacer-adjacent motif (PAM), CRISPR target, and *POMP*-ARE region are indicated. (D) Impact of *POMP*-ARE mutation on NRF3 recruitment. The indicated cells were subjected to ChIP assays using anti-NRF3 antibodies. Immunoprecipitated DNA was assessed by RT-qPCR assays using primers specific for the *POMP*-ARE region. ‡, *P* < 0.005; n.s., not significant (*n* = 3; means + SD) (determined by ANOVA followed by a Tukey test). (E and F) Impact of *POMP*-ARE mutation on mRNA and protein levels of POMP. mRNA and protein levels of POMP were assessed by RT-qPCR (E) and by immunoblotting (F), respectively. *, *P* < 0.05; n.s., not significant (*n* = 3; means + SD) (determined by ANOVA followed by a Tukey test [E]). (G) Impact of *POMP*-ARE mutation on proteasome activity. The indicated cell extracts were fractionated into 20 fractions by 10% to 40% glycerol gradient centrifugation and assayed for Suc-LLVY-AMC (chymotrypsin-like)-hydrolyzing activity of 20S proteasomes (+SDS/−ATP) (top) or 26S proteasomes (−SDS/+ATP) (bottom). The mean and individual values are represented as lines and marks, respectively (*n* = 2). The activity in fractions 1 to 5 was derived from nonproteasomal proteases.

The 20S proteasome is composed of two outer α- and two inner β-rings. The outer α-rings and the inner β-rings of the 20S proteasome consist of seven α-subunits (PSMA1 to -7) and seven β-subunits (PSMB1 to -7), respectively. The 19S-RP is further divided into “base” and “lid” subcomplexes, consisting of 6 subunits (PSMC1 to -6) and 13 subunits (PSMD1 to -4, PSMD6 to -8, PSMD11 to -14, ADRM1, and SHFM1), respectively. For most of these proteasome subunits, NRF3 overexpression did not increase mRNA levels (Fig. 2A); this was also found for several subunits, including PSMD7, PSMC5, PSMA1, and PSMB6, at the protein level (Fig. 2C). Similarly, NRF3 overexpression did not affect mRNA levels of four genes (*PSME1* to *PSME4*), which encode the subunits of an ATP-independent regulatory complex that, alternatively, binds the 20S proteasome instead of the 19S-RP (Fig. S2G). Meanwhile, among the five chaperones that promote 20S proteasome assembly, NRF3 significantly increased the mRNA and protein expression levels of POMP (Fig. 2B and C and 3E; Fig. S2B and H). POMP is a human ortholog of UMP1, which is found in precursor proteasomes with unprocessed β-subunits and is degraded upon the completion of proteasome assembly (14). This relationship suggests a role for NRF3 in the regulation of 20S proteasome assembly. To confirm the effects of NRF3 on proteasome assembly, we investigated the distributions of subunit proteins between control cells overexpressing green fluorescent protein (GFP) and NRF3 (Fig. 2D) and compared their protein levels in fraction 10 versus fraction 13 (Fig. 2E), where 20S and 26S proteasome activities were found, respectively (Fig. 1D). Interestingly, NRF3 overexpression led to significantly increased protein levels of the 20S proteasome subunits PSMA1 and PSMB6 in fraction 10 (Fig. 2E). On the other hand, the protein levels of the 19S-RP subunit PSMC5 in fraction 10 were not changed by NRF3 overexpression (Fig. 2E). This suggests that NRF3 positively regulates the assembly of 20S proteasomes in cancer cells by inducing the gene expression of its assembly chaperone *POMP* but not of the 20S proteasome subunits. Actually, *POMP* expression levels are increased in several cancer types (Fig. S1B). Moreover, NRF3 overexpression affected the PSMC5 protein distribution in fractions 3 to 5 and its protein reduction in fraction 13 (Fig. 2D and E), implying a negative role for NRF3 function in the regulation of 19S-RP and 26S proteasome assembly.

### NRF3 enhances 20S proteasome activity by directly inducing *POMP* gene expression.

NRF3 functions as a transcription factor by directly binding antioxidant response elements (AREs) (15). Chromatin immunoprecipitation (ChIP) sequencing analysis for global mapping of NRF3 binding sites in H1299 cells (our unpublished data) showed a positive peak at a conserved ARE sequence on the *POMP* gene (Fig. 3A). The presence of this NRF3 binding region near the transcription start site of the *POMP* gene was further validated by ChIP-quantitative PCR (qPCR) (Fig. 3B), establishing *POMP* as an NRF3 target gene. For direct evidence that NRF3 promotes 20S proteasome assembly through the induction of *POMP* expression, we generated *POMP*-ARE mutant cells through CRISPR/Cas9-based genome editing, using H1299-NRF3 overexpression cell clone 2 (H1299-oeNRF3#2) as the parental cell line (Fig. 3C), and obtained a mutant cell line with a thymine-to-guanine substitution in the *POMP*-ARE (here referred to as mtPOMP-oeNRF3#2). Comparative analyses of parental H1299-oeNRF3#2 cells and *POMP*-ARE mutant mtPOMP-oeNRF3#2 cells showed a reduced ChIP signal at the NRF3 binding site on the ARE region of the *POMP* gene in mtPOMP-oeNRF3#2 cells and reduced POMP expression at both the mRNA and protein levels, resulting in the complete suppression of 20S proteasome activity that was otherwise promoted by NRF3 overexpression (Fig. 3D to G). These findings provide evidence that NRF3 directly induces *POMP* gene expression, thereby enhancing 20S proteasome activity.

### NRF3 reduces Rb and p53 protein levels and abrogates p53-mediated tumor suppression signals.

**FIG 4:**
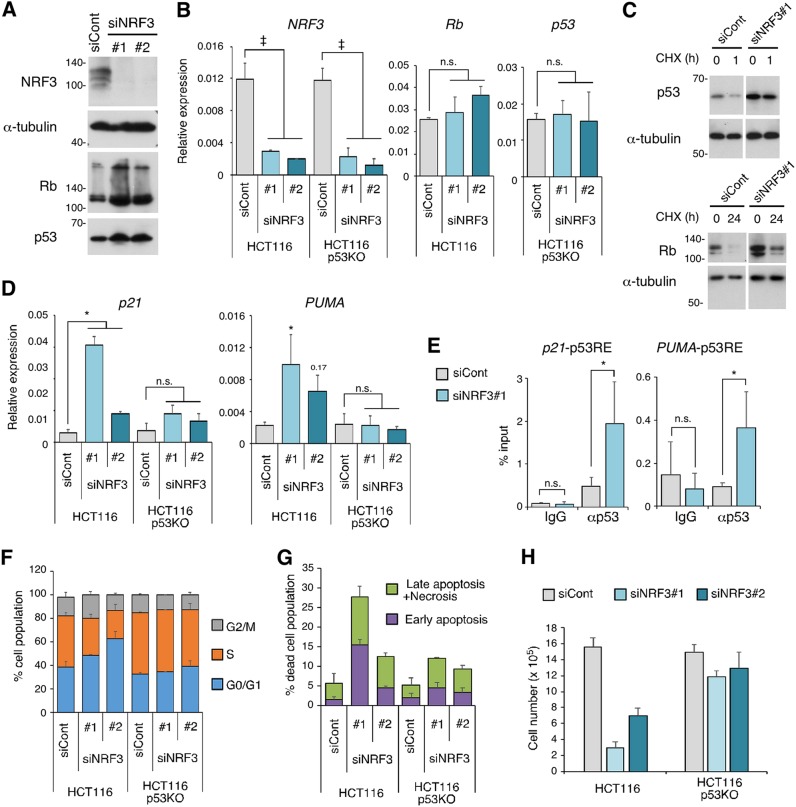
NRF3 knockdown increases Rb and p53 protein levels and induces p53-mediated cell cycle arrest and apoptosis. (A) Impact of NRF3 knockdown on Rb and p53 protein levels. HCT116 cells were transfected with the indicated siRNAs. After 2 days, the Rb and p53 proteins were detected by immunoblotting. (B) Impact of NRF3 knockdown on mRNA levels of *Rb* and *p53*. HCT116 cells were transfected with the indicated siRNAs. After 2 days, mRNA levels of the indicated genes were assessed by RT-qPCR. HCT116 p53KO cells were used as controls. ‡, *P* < 0.005; n.s., not significant (*n* = 3; means + SD) (determined by ANOVA followed by a Tukey test). (C) Impact of NRF3 knockdown on Rb and p53 protein levels under cycloheximide (CHX) treatment. HCT116 cells were transfected with the indicated siRNAs. Two days after transfection, the cells were treated with 50 μg/ml cycloheximide, and the whole-cell extracts were prepared at the indicated time points. (D) Impact of NRF3 knockdown on mRNA levels of two p53 target genes, *p21* and *PUMA*. HCT116 cells were transfected with the indicated siRNAs. After 2 days, mRNA levels of the indicated genes were assessed by RT-qPCR. HCT116 p53KO cells were used as controls. *, *P* < 0.05; 0.17, *P* = 0.17; n.s., not significant (*n* = 3; means + SD) (determined by ANOVA followed by a Tukey test). (E) Impact of NRF3 knockdown on p53 recruitment to the *p21* and *PUMA* promoters. HCT116 cells were transfected with the indicated siRNAs. After 2 days, ChIP assays were performed using anti-p53 antibodies, and immunoprecipitated DNA was assessed by RT-qPCR assays with primers specific for each p53 response element (p53RE). Normal mouse IgG was used as a control. *, *P* < 0.05; n.s., not significant (*n* = 3; means + SD) (determined by a *t* test). (F) Impact of NRF3 knockdown on p53-mediated cell cycle arrest. HCT116 cells were transfected with the indicated siRNAs. After 2 days, the cells were cultured with EdU for 2 h and stained with the Click-iT reaction mixture and PI following flow cytometry (*n* = 3; means + SD). HCT116 p53KO cells were used as controls. Representative contour plots are shown in Fig. S3A in the supplemental material. (G) Impact of NRF3 knockdown on p53-mediated apoptosis. HCT116 cells were transfected with the indicated siRNAs. After 2 days, the cells were stained with annexin V and PI, followed by flow cytometry (*n* = 3; means + SD). HCT116 p53KO cells were used as controls. The populations of annexin V-single-positive and annexin V-PI-double-positive cells are represented as “Early apoptosis” and “Late apoptosis + Necrosis,” respectively (Fig. S3C). (H) Impact of NRF3 knockdown on cell viability. NRF3 or control siRNA was transfected into the indicated cells. After 48 h, the cells were counted using a hemocytometer (*n* = 3; means + SD).

We investigated how NRF3 promotes cancer cell growth. Previous studies suggested that the 20S proteasome ubiquitin-independent pathway degrades several tumor suppressor proteins such as Rb and p53 (16). Interestingly, NRF3 knockdown increased both Rb and p53 protein levels without altering their mRNA levels (Fig. 4A and B; Fig. S3A). Moreover, cycloheximide (CHX) treatment experiments suggested that NRF3 knockdown increases the half-lives of p53 and Rb proteins (Fig. 4C). Thus, we focused on the effect of small interfering RNA (siRNA)-mediated transient NRF3 knockdown on the transcriptional activity and tumor suppression function of p53. As expected, NRF3 knockdown induced the expression of p53 target genes, including the cell cycle-inhibitory effector *p21* and the proapoptotic gene *PUMA* (Fig. 4D), and the recruitment of p53 protein to the *p21* and *PUMA* promoters (Fig. 4E). Moreover, NRF3 knockdown increased the proportion of G_0_/G_1_-phase cells and the percentage of apoptotic and/or necrotic cells in a p53-dependent manner (Fig. 4F and G; Fig. S3A and B). These results are consistent with the finding that cell growth inhibition by NRF3 knockdown depends on p53 activity (Fig. 4H). In addition, NRF3 knockdown inhibited the growth of p53-null H1299 and p53 mutant T98G cells as well as p53 wild-type HCT116, LNCaP, and A172 cells (Fig. 1C). Considering that p53 and Rb cooperatively suppress cancer development (1), these results suggest that NRF3 suppresses cell cycle arrest and apoptosis through protein degradation of Rb as well as p53.

The siRNA experiments showed that NRF3 knockdown induces p53 activation and cell death, suggesting that it is technically difficult to generate high-efficiency stable NRF3 knockdown cells using p53 wild-type HCT116 cells. Therefore, we generated NRF3 knockdown cells using p53-deficient (p53KO) HCT116 cells, demonstrating significant reductions of 20S proteasome activity and *POMP* expression in these cells (Fig. S3C to E, top). These results are consistent with the insignificant reduction of 20S proteasome activity and *POMP* expression in HCT116-NRF3 stable knockdown cells (Fig. 1D and Fig. 2B; Fig. S2B). We also observed reduced 26S proteasome activity in HCT116 p53KO-NRF3 stable knockdown cells (Fig. S3E, bottom). Considering that the 26S proteasome consists of the 20S proteasome and the 19S-RP, this result suggests that high-efficiency, stable NRF3 knockdown affects the assembly of the 26S proteasome as well as the 20S proteasome, although further study is needed to confirm this finding.

### The NRF3-POMP axis contributes to ubiquitin-independent proteolysis of Rb and p53 and resistance to the proteasome inhibitor anticancer agent BTZ.

**FIG 5:**
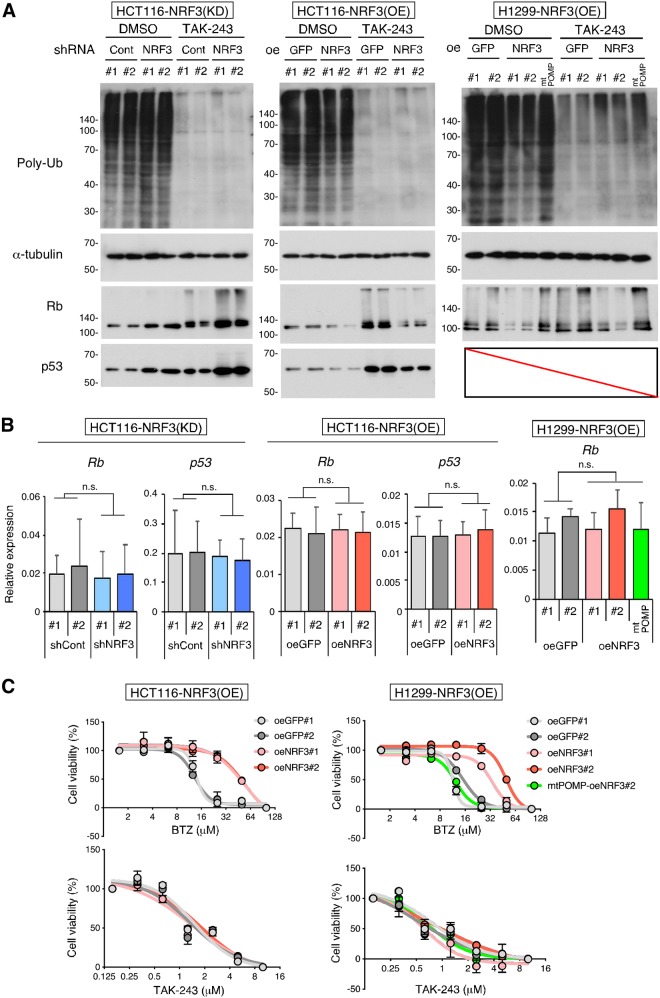
NRF3 contributes to the ubiquitin-dependent degradation of the Rb and p53 proteins and resistance to BTZ in a *POMP* gene expression-dependent manner. (A) Impacts of NRF3 and POMP on the ubiquitin (Ub)-independent degradation of the Rb and p53 proteins. Each protein was detected by immunoblotting after 24 h of treatment with 10 μM TAK-243, a ubiquitin-activating enzyme E1 inhibitor. DMSO was used as a control. *POMP*-ARE mutant mtPOMP-oeNRF3#2 cells were also used to check the impact of the defect on the NRF3-increased 20S proteasome. (B) Impacts of NRF3 and POMP on mRNA levels of *Rb* and *p53. Rb* and *p53* mRNA levels of the indicated stable cells were assessed by RT-qPCR. *POMP*-ARE mutant mtPOMP-oeNRF3#2 cells were also used to check the impact of the defect on the NRF3-increased 20S proteasome. n.s., not significant (*n* = 3; means + SD) (determined by ANOVA followed by a Tukey test). (C) Impacts of NRF3 and POMP on resistance to a proteasome inhibitor, BTZ, or a ubiquitin-activating enzyme E1 inhibitor, TAK-243. Viabilities of NRF3 overexpression HCT116 or H1299 cells were assessed by WST-1 assays after 24 h of treatment with the indicated concentrations of BTZ (top) or TAK-243 (bottom). *POMP*-ARE mutant mtPOMP-oeNRF3#2 cells were also used to check the defect’s impact on the NRF3-POMP axis (*n* = 3; means ± SD).

Previous insights into the ubiquitin-independent degradation of the Rb and p53 proteins were provided by *in vitro* assays (4, 5). In contrast, we sought to determine whether the NRF3-POMP axis affects the ubiquitin-independent degradation of endogenous Rb and p53. To address this issue, we examined the effects of a ubiquitin-activating enzyme E1 inhibitor, TAK-243 (17), on protein stability in cells. Irrespective of TAK-243 treatment, NRF3 knockdown increased the protein levels of Rb and p53, while NRF3 overexpression reduced the levels of these proteins (Fig. 5A; Fig. S3F, shCont versus shNRF3 or control oeGFP versus oeNRF3). However, *NRF3* knockdown or overexpression did not affect the mRNA levels of these proteins (Fig. 5B; Fig. S3G). More importantly, the *POMP*-ARE mutation impaired the NRF3-mediated reduction of Rb protein levels, irrespective of TAK-243 treatment (Fig. 5A, right, parental oeNRF3#2 versus *POMP*-ARE mutant mtPOMP-oeNRF3#2), suggesting that the upregulation of the NRF3-POMP axis leads to the ubiquitin-independent proteolysis of Rb and p53 in cancer cells. We also found that TAK-243 treatment increased endogenous Rb and p53 protein levels irrespective of NRF3 knockdown (Fig. 5A; Fig. S3F, control dimethyl sulfoxide [DMSO] versus TAK-243), indicating that both proteins are degraded via the NRF3-independent UPS as well as the NRF3-POMP-20S proteasome assembly axis.

The proteasome is a target for cancer chemotherapy, and several proteasome inhibitors have been developed as anticancer drugs (18). Thus, we investigated whether the NRF3-POMP axis affects the resistance of cancer cells to two distinct proteasome inhibitor anticancer agents: the proteasome inhibitor BTZ and the ubiquitin-activating enzyme E1 inhibitor TAK-243 (17, 19). The half-maximal inhibitory concentration (IC_50_) values for these drugs are shown in Table S2. NRF3 overexpression conferred resistance to BTZ but not TAK-243 (Fig. 5C). Furthermore, the *POMP*-ARE mutation abolished NRF3-mediated BTZ resistance (Fig. 5C, top right, parental oeNRF3#2 versus *POMP*-ARE mutant mtPOMP-oeNRF3#2), indicating that the upregulation of the NRF3-POMP axis diminishes the anticancer effects of BTZ but not those of TAK-243. These results are consistent with the direct inhibition of both 20S and 26S proteolytic activities by binding of BTZ to catalytic sites within the 20S proteasome subcomplex (20). Hence, we suggest that cancer cells acquire BTZ resistance through ubiquitin-independent proteolysis following the upregulation of the NRF3-POMP-20S proteasome assembly axis. The results obtained from NRF3 stable knockdown HCT116 p53KO cells were consistent with these findings (Fig. S3H, left). Meanwhile, sensitivity to these agents was not changed in NRF3 stable knockdown HCT116 cells (Fig. S3H, right), due to the insufficient reduction of 20S proteasome activity and *POMP* expression in these cells (Fig. 1D, top right, and Fig. 2B, right; Fig. S2B).

### Upregulation of the NRF3-POMP axis contributes to cancer development and poor prognosis.

**FIG 6:**
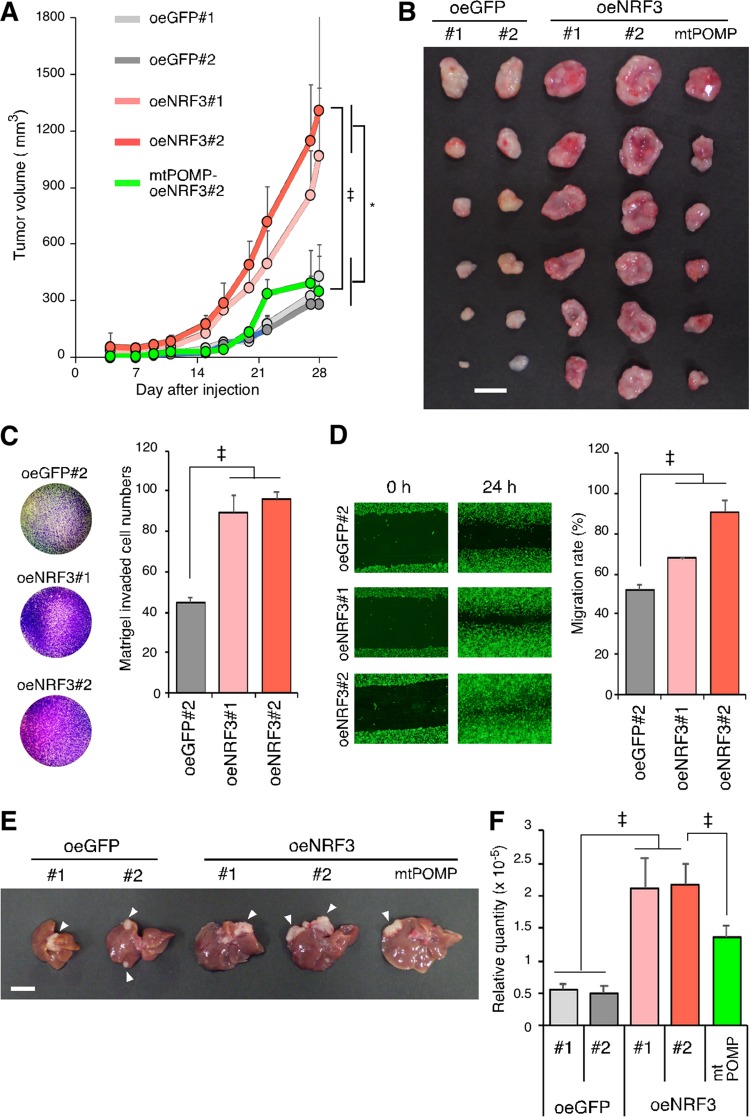
*POMP*-ARE mutation abolishes NRF3-induced tumor growth and hepatic metastasis. (A and B) Impacts of NRF3 and POMP on tumorigenesis. Mice were subcutaneously injected with the indicated H1299 cells in each flank. (A and B) Tumor growth curves (A) and photographs and weights of tumors 28 days after injection (B). *POMP*-ARE mutant mtPOMP-oeNRF3#2 cells were also used to check the defect’s impact on the NRF3-POMP axis. Bar, 10 mm. *, *P* < 0.05; ‡, *P* < 0.005 (*n* = 6; means + SD) (determined by ANOVA followed by a Tukey test [A]). (C and D) Impact of NRF3 overexpression on metastasis *in vitro*. The invasion and migration abilities of the indicated H1299 cells were assessed by transwell (C) and scratch (D) assays, respectively. GFP#2 cells were used as controls. ‡, *P* < 0.005 (*n* = 3; means + SD) (determined by ANOVA followed by a Tukey test). (E and F) Impacts of NRF3 and POMP on metastasis *in vivo*. Mice were injected in the spleen with the indicated H1299 cells. After 28 days, their livers were removed. Representative images (E) and RT-qPCR-based quantification (F) of hepatic metastasis are shown. Bar, 10 mm. Arrowheads indicate metastatic nodules. GFP-overexpressing cells were used as controls. *POMP*-ARE mutant mtPOMP-oeNRF3#2 cells were also used to check the defect’s impact on the NRF3-POMP axis. ‡, *P* < 0.005 (*n* = 5; means + SD) (determined by ANOVA followed by a Tukey test).

Next, we investigated the significance of the NRF3-POMP axis in tumorigenesis. Mouse xenograft experiments revealed that NRF3 overexpression results in increased tumor volume and weight, while the *POMP*-ARE mutation inhibited this effect (Fig. 6A and B). This suggests that NRF3 promotes tumor growth by enhancing 20S proteasome assembly through direct binding to the ARE of the *POMP* gene, promoting the upregulation of its expression. Recently, it was reported that pancreatic cancers with high NRF3 expression levels are more prone to lymph node metastasis (21), and it is known that Rb and p53 contribute to cancer metastasis (22). Therefore, we next investigated the significance of the NRF3-induced 20S proteasome on metastasis and found that NRF3 overexpression enhanced cancer cell invasion and migration *in vitro* (Fig. 6C and D). Furthermore, we performed an *in vivo* murine model of hepatic metastases via spleen injection of cancer cells (23). H1299-oeGFP, oeNRF3, and mtPOMP-oeNRF3#2 cells formed nodules on the mouse liver (Fig. 6E), indicating that all these cell lines qualitatively have metastatic potential. The number and size of nodules are generally considered metastatic indexes, although it is difficult to quantify exactly these indexes in the whole liver. Alternatively, we performed real-time qPCR (RT-qPCR) using primers specific for human *HPRT* that did not cross-react with the mouse homolog (24). In this assay, the metastatic efficiency is quantitatively represented by mRNA levels of the human *HPRT* gene derived from metastatic H1299 cells in the liver (see Materials and Methods for details). We confirmed that consistent with the above-described *in vitro* cell invasion and migration assay results, *NRF3* overexpression promoted hepatic metastasis progression (Fig. 6F, control oeGFP versus oeNRF3). More importantly, we showed that the *POMP*-ARE mutation partially, but significantly, reduced hepatic metastasis promoted by NRF3 overexpression (Fig. 6F, parental oeNRF3#2 versus *POMP*-ARE mutant mtPOMP-oeNRF3#2).

**FIG 7:**
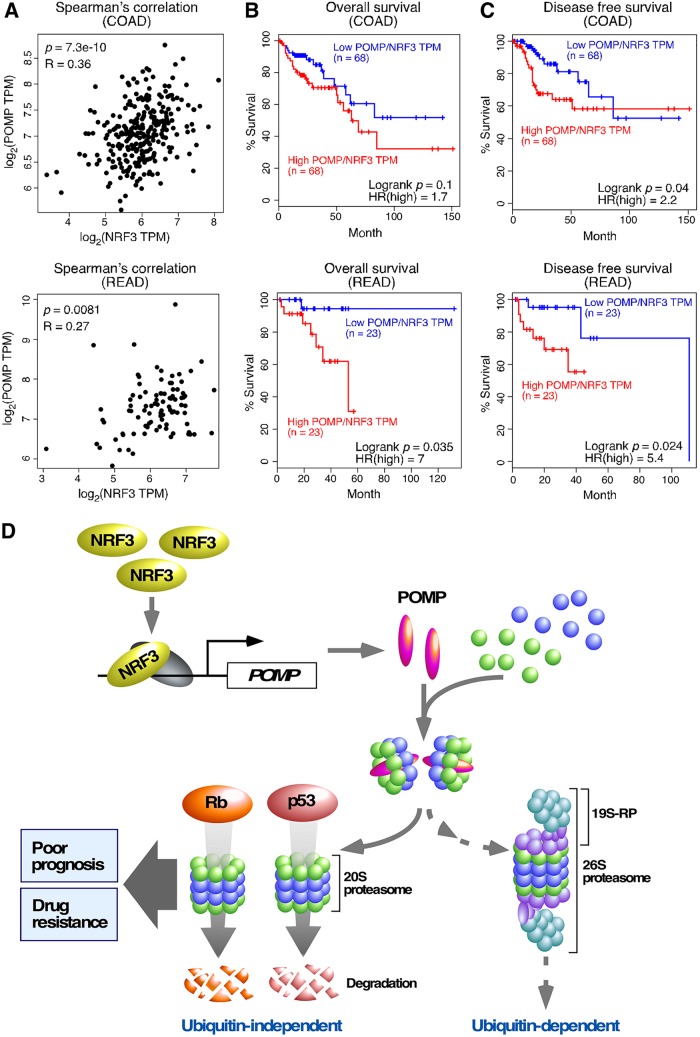
Colorectal cancer patients with higher *POMP/NRF3* expression levels exhibit poor prognoses. (A to C) Clinical association of *NRF3* and *POMP* genes with the prognoses of patients with colorectal adenocarcinoma (COAD) or rectal adenocarcinoma (READ). A Spearman correlation plot of these genes (A) and Kaplan-Meier analysis comparing overall (B) and disease-free (C) survival were analyzed using TCGA and GTEx data sets. The hazard ratio (HR) was calculated based on Cox’s proportional-hazards model. (D) Schematic model of cancer development through the NRF3-POMP-20S proteasome assembly axis. The *NRF3* gene is highly expressed in cancer cells, e.g., in colorectal adenocarcinoma. In these cancer cells, NRF3 transcribes the *POMP* gene and indirectly assembles the 20S proteasome. Upregulation of the NRF3-POMP-20S proteasome assembly axis degrades Rb and p53 in a ubiquitin-independent manner, thereby abrogating the tumor suppression signals, including cell cycle arrest and apoptosis. Cancer patients with tumors expressing higher levels of *POMP/NEF3* exhibit lower overall and disease-free survival rates. NRF3-expressing cancer cells also develop resistance to BTZ-type proteasome inhibitor anticancer drugs, which directly bind to the catalytic sites within the 20S proteasome subcomplex.

Finally, we validated the relevance of the NRF3-POMP axis in a clinical setting. Recently, *NRF3* gene amplification was reported in colorectal cancer patients (25). We also observed that *NRF3* mRNA is increased in intestinal tissues derived from *Apc*-mutated mouse and intestine-derived mouse organoids with *Apc* gene deletion, both of which are well-established colorectal cancer models (26). Here, we first confirmed the positive correlation between *NRF3* expression and *POMP* expression in patients with colorectal adenocarcinoma (COAD) or rectal adenocarcinoma (READ) (Fig. 7A). In addition, we showed that higher *POMP*/*NRF3* mRNA levels correlated significantly with lower overall survival and lower disease-free survival rates of these cancer patients (Fig. 7B and C), corroborating the importance of the NRF3-POMP axis for cancer development and colorectal cancer prognosis.

## DISCUSSION

Aberrant proteolytic activity is a hallmark of cancer and occurs without genetic mutation of the proteolytic machinery (2). The regulation and function of the UPS have been researched extensively, while those of the ubiquitin-independent 20S proteasome system remain obscure. In this study, we revealed that the NRF3-POMP axis contributes to cancer cell growth through ubiquitin-independent proteolysis of tumor suppressors (Fig. 7D). In cancer cells, NRF3 induces *POMP* gene expression and enhances 20S proteasome assembly. The NRF3-POMP-20S proteasome assembly axis contributes to the protein degradation of Rb and p53 in a ubiquitin-independent manner, leading to tumorigenesis and metastasis. Furthermore, upregulation of the axis causes resistance to BTZ-type anticancer drugs and is correlated with poor prognoses of colorectal cancer patients. Although a previous study showed that microRNA 101 (miR101) inhibits POMP-mediated assembly of the 20S proteasome and suppresses tumor growth (27), the *POMP*-ARE mutation assay results in this study indicate that the upregulation of the NRF3-POMP axis enhances 20S proteasome assembly and promotes tumorigenesis in an miR101-independent manner. Thus, our present study sheds light on the impact of 20S proteasome regulation and function on cancer cells and provides crucial insights into three related issues, (i) the inactivation of the tumor suppressors p53 and Rb in cancer cells, (ii) cross talk of NRF3 with the homologs NRF1 and NRF2, and (iii) the development of anticancer drugs, as discussed in more detail below.

(i) The tumor suppressors p53 and Rb are inactivated by the UPS and cyclin-dependent kinase (Cdk) phosphorylation, respectively (28, 29). In the absence of inactivation, p53 and Rb can cooperatively suppress cancer development (1). For example, p53-null and Rb-null mice each generally show normal cell and tissue homeostasis yet develop abnormalities later in life (30). Meanwhile, cells lacking both *p53* and *Rb* show telomere dysfunction, leading to chromosomal end-to-end joining and fusion-bridge-breakage cycles that can trigger the aneuploidy observed in most cancers (31). Furthermore, dual inactivation of p53 and Rb functions is essential for suppressing oncogenic Ras-induced melanocyte transformation *in vivo* (32). Hence, we suggest that both the p53 and Rb proteins are potential substrates for the NRF3-dependent ubiquitin-independent 20S proteasome system as well as for the NRF3-independent UPS (Fig. 5A; Fig. S3F), although our present results do not exclude the possibility that the axis affects other 20S proteasome target proteins, as reported previously (16). A previous study showed that a stress-inducible flavoprotein, NQO1, interacts with p53 and inhibits the ubiquitin-independent proteolysis of p53 in the presence of NADH (4). Meanwhile, an E3 ubiquitin ligase for p53, MDM2, is required for the ubiquitin-independent proteolysis of Rb (5). These insights imply that the protein levels of p53 and Rb might be coordinated by the NRF3-dependent ubiquitin-independent 20S proteasome system and/or the NRF3-independent UPS in response to cellular metabolic status.

(ii) A human genome study showed that *NRF2* and *NRF3* are among 127 significantly mutated genes (SMGs) across 12 major cancer types (11), breast adenocarcinoma (BRCA), lung adenocarcinoma (LUAD), acute myeloid leukemia (LAML; conventionally called AML), lung squamous cell carcinoma (LUSC), uterine corpus endometrial carcinoma (UCEC), bladder urothelial carcinoma (BLCA), COAD, glioblastoma (GBM), head and neck squamous cell carcinoma (HNSC), kidney renal clear cell carcinoma (KIRC), ovarian cancer (OV), and READ, with abundant *NRF3* mRNA expression (Fig. 1A, top). Although *NRF1* is not among the SMGs, we found that *NRF1* mRNA is abundant in both tumor and normal specimens (Fig. 1A, bottom). NRF1 activates the gene transcription of both the 20S proteasome and 19S-RP subunits in response to proteasome inhibition (8–10). Meanwhile, NRF2 activates the transcription of these genes in response to oxidative stress and in the presence of a p53 missense mutation (33, 34). Interestingly, both NRF1 and NRF2 induce *POMP* gene expression in response to treatment with proteasome inhibitors (8–10, 35). We recently found that NRF3 also induces the expression of several proteasome-related genes upon proteasome inhibition (our unpublished data). These findings suggest a possible transcriptional network constituted by the three NRF forms, which cooperatively and/or competitively orchestrate the balance between the 20S and 26S proteasomes in cancers such as COAD and READ. Indeed, we have already revealed cross talk between NRF1 and NRF3 (T. Waku, H. Katayama, M. Hiraoka, A. Hatanaka, N. Nakamura, Y. Tanaka, N. Tamura, A. Watanabe, A. Kobayashi, submitted for publication). Furthermore, NRF3 may partly utilize the same target genes as NRF2 as well as NRF1 to promote tumor formation, because the amino acid sequences of the DNA binding domain are similar among NRF1, NRF2, and NRF3.

(iii) Inhibition of proteasome function has emerged as a powerful strategy for anticancer therapy (18). However, treatment of cancer patients with BTZ increases the risk of neuropathy and causes nausea, diarrhea, and fatigue because the proteasome is indispensable for the functioning of both cancer and normal cells (18). Therefore, we propose NRF3 inhibitors as alternative anticancer drugs that could reduce and/or avoid the serious adverse effects of BTZ treatment; we believe that this is likely because of the lack of any apparent abnormalities in *NRF3* knockout mice under normal physiological conditions (36, 37). To inhibit NRF3 function, we focus on the aspartic protease DDI2, which cleaves and activates NRF3 (7). DDI2 inhibition could therefore supplant direct proteasome inhibitors like BTZ. Fortuitously, structural studies have identified a retroviral protease-like domain in DDI2 similar to the HIV-1 protease (HIVp), which is a therapeutic target for HIV (38). This suggests that the HIVp inhibitors used in HIV treatment could inhibit DDI2-mediated activation of NRF3, abrogating aberrant 20S proteasome activity in cancer cells. Indeed, it has been reported that HIVp inhibitors, including ritonavir, nelfinavir, and saquinavir, attenuate proteasome activity and inhibit cancer cell growth *in vitro* (39). Nelfinavir, synergistically with BTZ, further induces the proteotoxic death of cancer cells *in vivo* (40). Although further study is needed to confirm whether specific inhibition of the 20S proteasome assembled by the NRF3-POMP axis reduces cancer development, our findings shed light on the potential to reposition anti-HIV agents, such as HIVp inhibitors, as anticancer drugs in order to inhibit DDI2 and indirectly inhibit the tumorigenic function of NRF3.

## MATERIALS AND METHODS

### Cell lines and antibodies.

Wild-type HCT116, p53-deficient (p53KO) HCT116 (41), A-172, T98G, and HeLa cells were cultured in Dulbecco’s modified Eagle’s medium (DMEM)–high-glucose medium (Wako Pure Chemical Industries). H1299 and LNCaP cells were cultured in RPMI 1640 medium (Nacalai Tesque). U2OS cells were cultured in DMEM–high-glucose medium with 2 mM l-glutamine. All media were supplemented with 10% fetal bovine serum (FBS) (Nichirei Biosciences), 40 μg/ml streptomycin, and 40 U/ml penicillin (Life Technologies).

Antibodies against the proteins α-tubulin (clone DM1A; Sigma-Aldrich), p53 (clone DO-1, catalog number sc-126; Santa Cruz Biotechnology), retinoblastoma (catalog number sc-102; Santa Cruz Biotechnology), polyubiquitin (clone P4D1, catalog number sc-8017; Santa Cruz Biotechnology), and GFP (catalog number sc-9996; Santa Cruz Biotechnology) and unconjugated affinity-purified isotype control immunoglobulin G (IgG) from mouse (catalog number sc-2025; Santa Cruz Biotechnology) were used in this study. Anti-human NRF3 antibodies (clone number 9408) were reported previously (7). Antibodies against the following proteasome-related proteins were generated previously: PSMA1 (α6) and PSMC5 (Rpt6) (42), PSMB6 (β1) (43), PSMD6 (Rpn8) (44), and POMP (UMP1) (45).

### Generation of NRF3 overexpression or knockdown cell lines.

To generate stable overexpressing cell lines, H1299 or HCT116 cells were transfected with the p3XFLAG-CMV10 vector (Sigma-Aldrich) containing full-length human NRF3 or GFP. The transfected cells were selected with G-418. To generate stable knockdown cell lines, HCT116 or HCT116 p53KO cells were transfected with the piGENE hU6 plasmid (iGENE Therapeutics) containing human NRF3 target or control sequences (see Table S3 in the supplemental material). The transfected cells were selected with puromycin.

### Genome editing of the POMP-ARE using the CRISPR/Cas9 system.

To edit the ARE-like sequence in the *POMP* promoter, NRF3-overexpressing H1299 (H1299-oeNRF3#2) cells were cotransfected with pEGFP-N1 (Clontech) and pX330-U6-Chimeric_BB-CBh-hSpCas9 (Addgene plasmid 42230) expressing guide RNA for the *POMP*-ARE. After 24 h, GFP-positive cells were sorted using a flow cytometer (FACSAria II; BD Biosciences). Finally, the cell line with the *POMP*-ARE mutation was selected by genomic DNA sequencing.

### RNA extraction, cDNA synthesis, and real-time quantitative PCR.

Total RNA was extracted and purified using Isogen II (Nippon Gene) according to the manufacturer's instructions. Aliquots of total RNA (1 μg) were reverse transcribed using pd(N)6 random primer (Takara Bio) and Moloney murine leukemia virus (M-MLV) reverse transcriptase (Invitrogen) with 250 μM deoxynucleoside triphosphate (dNTP; Takara Bio), according to the manufacturer’s instructions. Real-time quantitative PCR (RT-qPCR) was performed with SYBR Ex *Taq* II premix (TaKaRa Bio) and gene primers (Table S3) using Dice real-time thermal cycler system II (TaKaRa Bio). The relative expression level of each gene was normalized to the mRNA levels of the β-actin gene.

### Immunoblot analysis.

To prepare whole-cell extracts, the cells were lysed with SDS sample buffer (50 mM Tris-HCl [pH 6.8], 10% glycerol, and 1% SDS) containing a protease inhibitor cocktail (PIC) (Nacalai Tesque). For immunoblotting using TAK-243 (catalog number MLN7243; Active Biochem), cells were lysed with radioimmunoprecipitation assay (RIPA) buffer (25 mM Tris-HCl [pH 7.6], 150 mM NaCl, 1% NP-40, 1% sodium deoxycholate, and 0.1% SDS) containing protease inhibitors.

### Chromatin immunoprecipitation-qPCR and sequencing.

For quantification of NRF3 binding to the target regions, RT-qPCR was performed using purified DNA with the primers listed in Table S3. For chromatin immunoprecipitation (ChIP) sequencing, the libraries were prepared from 500 pg of immunoprecipitated DNA fragments using the Kapa hyperprep kit (Kapa Biosystems). The libraries were applied for single-end sequencing for 93 cycles on the HiSeq 2500 platform (Illumina). All sequence reads were extracted in FASTQ format using BCL2FASTQ 1.8.4 conversion software in the CASAVA 1.8.2 pipeline. Mapping was performed by BWA (version 0.5.9rc1) using the reference human genome of NCBI build 37 (hg19), and peak calling was conducted using MACS (version 1.4.2).

### Immunocytochemical staining.

Cells were seeded onto 6-well plates and transfected with siRNAs using RNAiMAX reagent (Invitrogen). After 24 h, the cells were fixed with 4% paraformaldehyde for 15 min at room temperature, washed three times with 0.1% PBS-T (0.1% Triton X-100 in phosphate-buffered saline [PBS]), permeabilized with 0.5% Triton X-100 for 10 min at room temperature, and washed twice with PBS-T. After treatment with blocking solution (1% skim milk in PBS-T) for 1 h at room temperature, the cells were incubated with anti-p53 or anti-Rb antibodies for 1 h at room temperature, washed three times with PBS-T, and incubated with Alexa Fluor 488- or Alexa Fluor 546-conjugated secondary antibodies along with 4′,6-diamidino-2-phenylindole (DAPI) for 2 h at room temperature. Finally, the samples were washed twice with PBS-T followed by PBS and were placed onto glass slides containing a drop of fluorescent mounting medium (Dako). Fluorescence images were captured by using a Zeiss LSM710 confocal microscope.

### Glycerol density gradient centrifugation and fluorogenic peptidase assays.

Glycerol density gradient centrifugation was conducted as described previously (46). After centrifugation at 26,000 rpm for 22 h in a Beckman SW40 Ti swing rotor, the gradient was manually separated into 20 fractions of 500 μl each. Thirty microliters of each fraction sample was transferred to a 96-well BD Falcon microtiter plate (BD Biosciences) and mixed with 0.1 mM fluorogenic peptide substrate. To measure chymotrypsin-like proteasome activity, we used either succinyl-Leu-Leu-Val-Tyr-7-amino-4-methylcoumarin (Suc-LLVY-AMC) (Peptide Institute) or benzyloxycarbonyl-Gly-Gly-Leu-7-amido-4-methylcoumarin (Z-GGL-AMC) (Enzo Life Sciences). Benzyloxycarbonyl-Leu-Leu-Glu-7-amido-4-methylcoumarin (Z-LLE-AMC) and acetyl-Arg-Leu-Arg-7-amido-4-methylcoumarin (Ac-Arg-Leu-Arg-AMC) (both Enzo Life Sciences) were used to measure the caspase-like and trypsin-like proteasome activities, respectively. Fluorescence (380-nm excitation and 460-nm emission) was monitored on a microplate fluorometer (Synergy HTX; BioTek Instruments) every 5 min for 1 h. To measure 20S or 26S proteasome activity, 0.05% SDS or 2 mM ATP was added to the mixture (13). Proteasome activity was calculated as the fluorescence intensity change over time (Δ*F*) using the Microsoft Excel slope function.

### Mouse xenograft and hepatic metastatic models.

Mouse xenograft models were used based on a previous report (47). Each 4-week-old BALB/cA-nu castrated female mouse (CLEA Japan) was injected subcutaneously with 100 μl of the cell suspension (8 × 10^6^ cells). Tumor growth was monitored by measuring the tumor size using calipers. Tumor volume was determined using the formula *V* = 1/2 × larger diameter × (smaller diameter)^2^. Mouse hepatic metastatic assays by splenic injection were performed based on a previous report (23). The spleen of each 4-week-old BALB/cA-nu castrated female mouse (CLEA Japan) was injected with the indicated cell lines (5 × 10^5^ cells). Hepatic metastases were quantified by RT-qPCR using primers specific for human *HPRT* that did not cross-react with the mouse homolog. 18S rRNA was used for normalization (24). The primer sequences are detailed in Table S3. All animal experiments were performed in accordance with the guidelines for the care and use of laboratory animals of Doshisha University, Japan.

### *In vitro* invasion and migration assays.

For the transwell invasion assay, 1 × 10^5^ cells were suspended in serum-free culture medium. Falcon cell culture inserts (24-well inserts with a membrane pore size of 8 μm; Corning) were used, and the cell suspension was placed on the top chamber precoated with Matrigel (Corning). Culture medium supplemented with 10% FBS was used as the chemoattractant in the bottom chamber. After 24 h, cells on the lower surface of the membrane were fixed with 70% ethanol and stained with 0.05% crystal violet. The stained invasion areas were measured using ImageJ software. For the scratch migration assay, the cells were cultured to confluence in 12-well plates and scratched with a plastic tip. After 24 h, the scratched migration areas were measured using the MRI Wound Healing Tool ImageJ macro.

### Cell cycle assay.

The cell cycle assay was conducted using a Click-iT EdU imaging kit (Invitrogen), according to the manufacturer’s protocol. Cells were seeded onto 6-well plates and transfected with siRNAs using RNAiMAX reagent (Invitrogen). After 2 days, the cells were labeled with 10 μM EdU (5-ethynyl-2′-deoxyuridine) in culture medium for 2 h at 37°C and collected in a microtube. The cell pellets were fixed with Click-iT fixative (containing paraformaldehyde) for 15 min at room temperature, followed by washing twice with 1% bovine serum albumin (BSA) in PBS and permeabilization with P/W buffer (1× Click-iT saponin-based permeabilization-and-wash reagent) for 15 min at room temperature. After treatment with the Click-iT reaction mixture for 30 min at room temperature in the dark, the cells were washed with P/W buffer and stained with propidium iodide (PI) for 20 min at 37°C in the dark. Finally, the cells were washed twice with P/W buffer and subjected to cell cycle analysis using a flow cytometer (FACSAria II; BD Biosciences).

### Dead-cell assay.

Dead-cell assays were conducted using an Alexa Fluor 488-annexin V/dead-cell apoptosis kit (Invitrogen) according to the manufacturer’s protocol. Cells were seeded onto 6-well plates and transfected with siRNAs using RNAiMAX reagent (Invitrogen). After 2 days, the cells were collected and resuspended in 1× annexin binding buffer with an anti-annexin V antibody conjugated with Alexa Fluor. Following incubation for 15 min at room temperature, the mixture was further stained on ice with PI. Finally, the cells were subjected to dead-cell analysis using a flow cytometer (FACSAria II; BD Biosciences). Annexin V-single-positive (early apoptotic) and annexin V-PI-double-positive (late apoptotic/necrotic) cells were considered dead cells in this study.

### WST-1 cell viability assay.

For the WST-1 assay, cells were seeded onto 96-well plates. After 24 h, BTZ (Peptide Institute) or TAK-243 was added, and the cells were further incubated for 24 h. Next, the cells were incubated with WST-1 reagent [2-(4-iodophenyl)-3-(4-nitrophenyl)-5-(2,4-disulfophenyl)-2H-tetrazolium, monosodium salt] (Nacalai Tesque) for 30 min. The absorbance was measured at 450 nm using a microplate reader (Synergy HTX; BioTek Instruments). IC_50_ values with statistics were calculated using GraphPad Prism 7 software and are summarized in Table S2.

### Cell fractionation.

Cells were suspended in buffer A (20 mM Tris-HCl [pH 8.0], 10 mM KCl, 1.5 mM MgCl_2_, 0.1 mM EDTA, 1 mM dithiothreitol [DTT], protease inhibitor cocktail [Nacalai Tesque]), followed by lysis by the addition of NP-40 (final concentration of 2.5%). After flash centrifugation at 10,000 rpm, the supernatants and precipitates were separated. The supernatants were further subjected to centrifugation at 20,000 × *g* for 10 min, and the resultant supernatants were utilized as cytoplasmic extracts. For the preparation of nuclear extracts, the precipitates were washed two times with buffer A and lysed with SDS sample buffer under mild sonication to shear genomic DNA. After centrifugation, the supernatants were collected as nuclear extracts.

### Cycloheximide treatment.

HCT116 cells were transfected with the indicated siRNAs. Two days after transfection, the cells were treated with 50 μg/ml cycloheximide (CHX), and whole-cell extracts were prepared at the indicated time points. Immunoblot analysis was conducted with the indicated antibodies.

### Statistics and human cancer data sets.

Unpaired Student’s *t* test was used to compare two groups, and one-way analysis of variance (ANOVA) followed by Tukey’s *post hoc* test was used to compare multiple groups. Gene Expression Profiling Interactive Analysis (GEPIA) is an online tool for analyzing RNA sequencing expression data from The Cancer Genome Atlas (TCGA) and the Genotype-Tissue Expression (GTEx) projects (48). GEPIA was used for dot plot profiling in Fig. 1A, the Spearman correlation plot in Fig. 7A, and Kaplan-Meier analyses in Fig. 7B and C. Abbreviations of cancer types shown in Fig. 1A and Fig. S1B are summarized with the numbers of specimens in Table S1.

## Supplementary Material

Supplemental file 1
