# Supplementary material for: NRF3-POMP-20S Proteasome Assembly Axis Promotes Cancer Development via Ubiquitin-Independent Proteolysis of p53 and Retinoblastoma Protein
Source: Mol Cell Biol. 2020 Apr 28;40(10):e00597-19. doi: 10.1128/MCB.00597-19 (PMC7189095; doi:10.1128/MCB.00597-19)
Supplement: Supplemental file 1 [file MCB.00597-19-s0001.pdf]

Supplementary Figure 1. *NRF* hotspot mutation and *POMP* expression levels in cancers (**A**) Lollipop plots of mutations in *NRF2* (top) and *NRF3* (bottom). Somatic mutation data from TCGA PanCancer Atlas studies, including 33 tumor types (10976 samples), were analyzed at the cBioPortal for Cancer Genomics (http://cbioportal.org). (**B**) Dot plots showing *POMP* gene expression across multiple cancer types and paired normal samples. Red and green dots represent RNA sequencing expression values of patient-matched tumors and adjacent normal tissue archived at TCGA and GTEx database. Red and blue abbreviations at the upper part of each graph indicate a cancer type with significantly higher and lower expression levels of each *NRF* gene compared to normal samples, respectively (TPM, transcripts per millions; ANOVA, q value cutoff = 0.01). Abbreviations of cancer types were summarized with the numbers of specimens in Supplementary Table 1.

Supplementary Figure 2. NRF3 induces POMP gene expression and enhances 20S proteasome activity. (**A**) Endogenous NRF3 protein levels in HCT116 (colorectal carcinoma), H1299 (non-small cell lung cancer), LNCaP (prostate adenocarcinoma), A-172 (glioblastoma), T98G (glioblastoma multiforme), U2OS (bone osteosarcoma) and HeLa (cervical adenocarcinoma) cell lines. Endogenous NRF3 protein levels were detected by immunoblotting. (**B**) Generation of NRF3 knockdown or overexpression cells using HCT116 or H1299 cells. NRF3 and POMP protein levels were detected by immunoblotting. GFP was used as control. (**C**) NRF3 protein levels in nucleus of H1299-oeNRF3#2 or oeGFP#2 cells. Each cell line was fractionated into the cytoplasmic (C) and nuclear extracts (N), followed by immunobloting. Whole cell extracts were used as input samples. (**D**) Impact of SDS or ATP on proteasome activity. HCT116 cell extracts were fractionated into 20 fractions, using a 10%–40% glycerol gradient centrifugation, and assayed for Suc-LLVY-AMC (chymotrypsin-like) hydrolysing activity of 20S proteasomes (+SDS/−ATP, black) or 26S proteasomes (−SDS/+ATP, gray). (N = 1) (**E**) Impact of a proteasome inhibitor MG-132 or a protease inhibitor cocktail (PIC) on protease or proteasome activity. HCT116 cell extracts were fractionated into 20 fractions, using a 10%–40% glycerol gradient centrifugation, and assayed for Suc-LLVY-AMC (chymotrypsin-like) hydrolysing

activity under treatment with 1 × PIC (red) (Nacalai Tesque) or 10 µM MG-132 (blue) (Peptide Institute) in the presence of SDS and the absence of ATP. (N=1) (**F**) Impact of NRF3 overexpression on three types of 20S proteasome activity. The indicated H1299 cell extracts used in Fig. 1D were assayed for Z-GGL-AMC (chymotrypsin-like), Ac-RLR-AMC (trypsin-like), and Z-LLE-AMC (caspase-like) hydrolysing activity of 20S proteasomes (+SDS/-ATP). Mean and individual values are represented as lines and marks, respectively (N = 2). The activity in fractions #1–#5 was derived from non-proteasomal proteases. (**G**) Impact of NRF3 overexpression on mRNA levels of four ATP-independent regulatory complex subunits. mRNA levels of indicated genes in H1299-oeNRF3#2 or oeGFP#2 cells were represented as red or blue bars, respectively. H1299-GFP#2 cells were used as controls. *p < 0.05; n.s., not significant (N = 3, mean ± SD, t-tests) (**H**) Impact of NRF3 overexpression on mRNA levels of four 20S proteasome assembly chaperones *PSMG1–4*. Each *PSMG* mRNA levels H1299-oeNRF3#2 or oeGFP#2 cells were represented as red or blue bars, respectively. H1299-GFP#2 cells were used as controls. *p < 0.05 (N = 3, mean ± SD, ANOVA followed by Tukey test)

Supplementary Figure 3. Impact of NRF3 knockdown in HCT116 p53KO cells on cell fate, POMP expression, proteasome activity and drug resistance. (**A**) Impact of NRF3 knockdown on Rb and p53 protein levels. HCT116 cells were transfected with indicated siRNA. After 2 d, Rb and p53 proteins were detected by immunostaining. HCT116 p53KO cells were used as controls. Scale bar, 10 µm. (**A** and **B**) Representative contour plots of cell-cycle assay in Fig. 4F (**A**) and cell-cycle assay in Fig. 4G (**B**). (**C**) Generation of NRF3 knockdown cells using HCT116 p53KO cells. NRF3 and POMP proteins were detected by immunoblotting. (**D**) Impact of NRF3 knockdown on mRNA levels of *POMP* in HCT116 p53KO cells. *POMP* mRNA levels were assessed by RT-qPCR. *p < 0.05 (N = 3, mean ± SD, ANOVA followed by Tukey test) (**E**) Impact of NRF3 knockdown on proteasome activity in HCT116 p53KO cells. The indicated cell extracts were fractionated into 20 fractions, using a 10%–40% glycerol gradient centrifugation, and assayed for Suc-LLVY-AMC (chymotrypsin-like) hydrolysing activity of 20S proteasomes (+SDS/−ATP, top) or 26S proteasomes (−SDS/+ATP, bottom). Mean and individual values are

represented as lines and marks, respectively ($N = 2$). The activity in fractions #1–#5 was derived from non-proteasomal proteases. (**F**) Impact of NRF3 knockdown on the ubiquitin-independent degradation of Rb and p53 proteins in HCT116 p53KO cells. Each protein was detected by immunoblotting after 24 h of treatment with 10 μM TKA-243, a ubiquitin activating enzyme E1 inhibitor. DMSO was used as the control. (**G**) Impact of NRF3 knockdown on mRNA levels of *Rb* in HCT116 p53KO cells. *Rb* mRNA levels were assessed by RT-qPCR. *$p < 0.05$ ($N = 3$, mean ± SD, ANOVA followed by Tukey test) (**H**) Impact of NRF3 knockdown on the resistance to proteasome inhibitor anticancer agents of HCT116 p53KO cells (left) or HCT116 cells (right). Viabilities of indicated cells were assessed by WST-1 assays after 24 h of treatment with the indicated concentration of BTZ (top) or TAK-243 (bottom) ($N = 3$, mean ± SD).

Waku et al., Revised Supplementary Figure 1

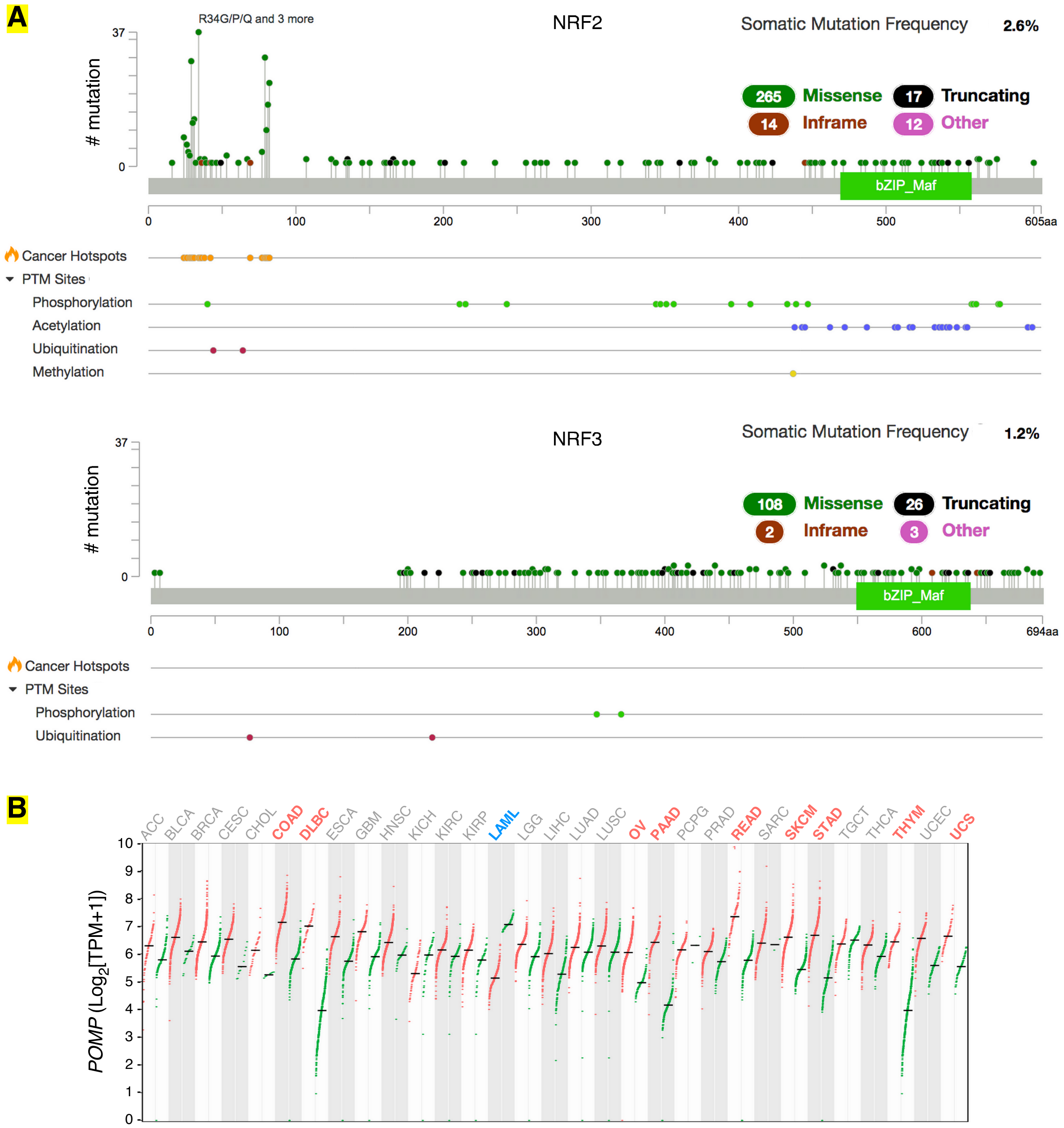

Waku et al., Revised Supplementary Figure 2

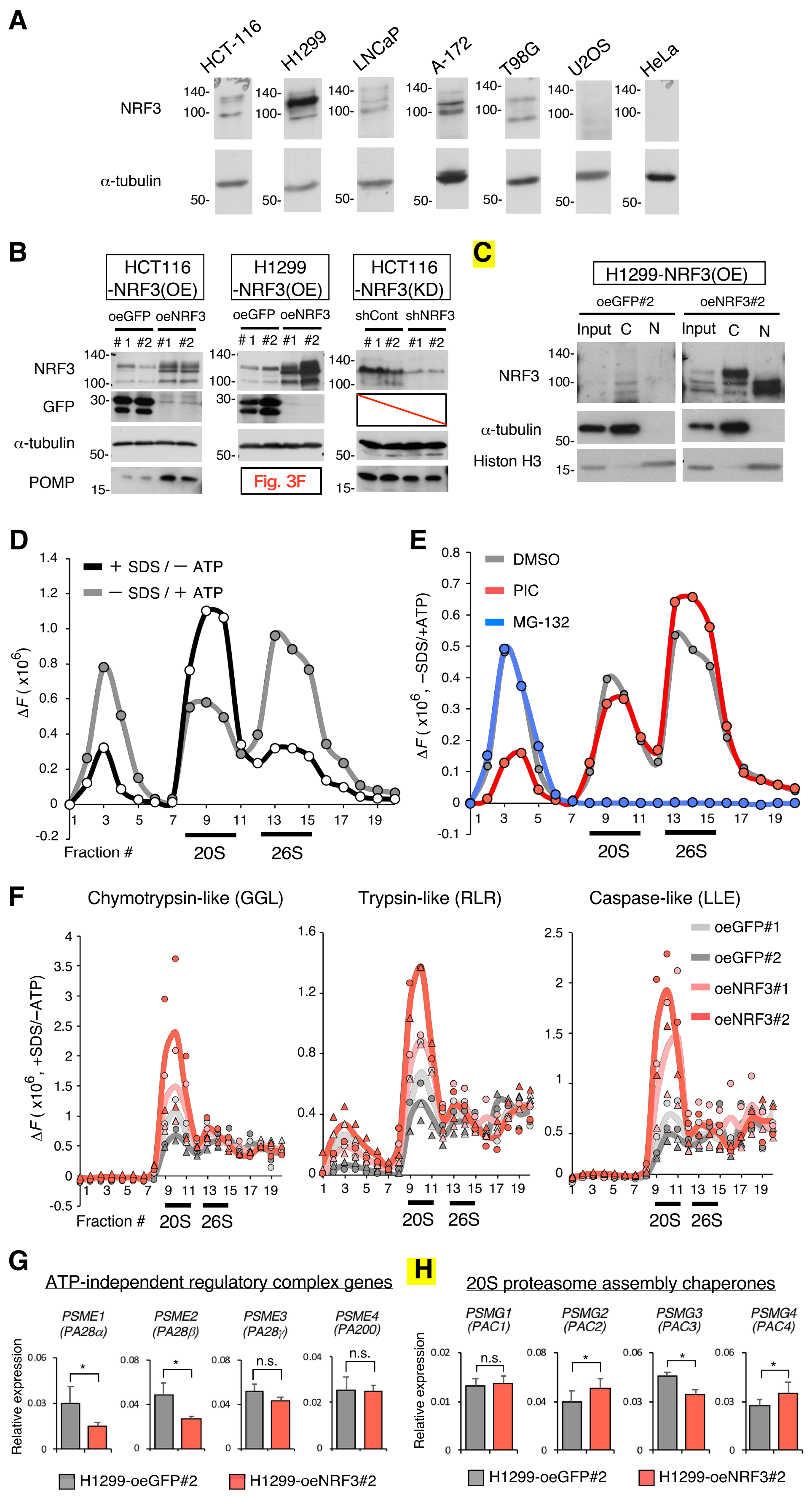

Waku et al., Revised Supplementary Figure 3

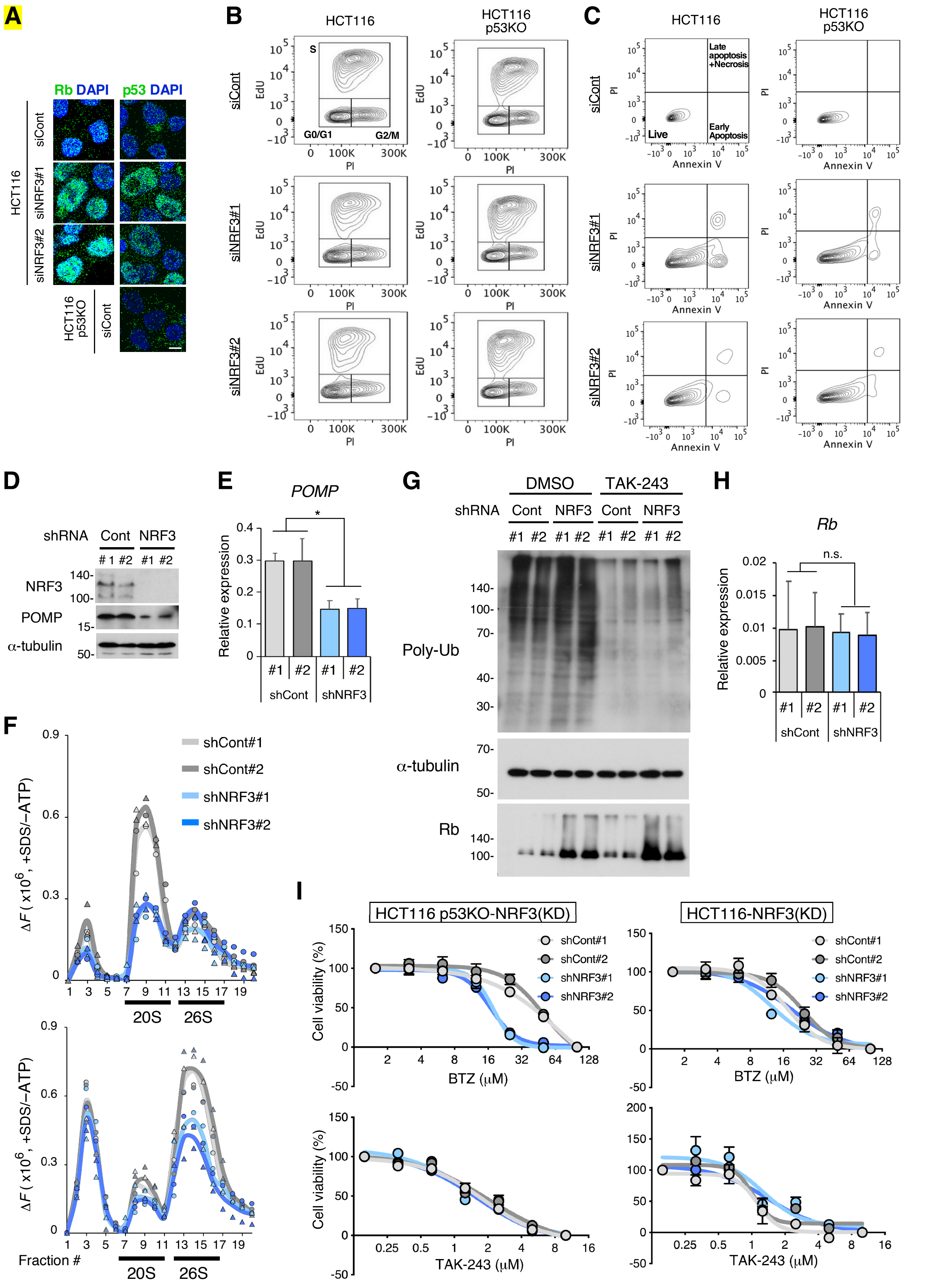

Revised Supplementary Table 1. Abbreviations of cancer types and the numbers of specimens analyzed

Abbreviation	Cancer types	Numbers of specimens (Tumor / Normal)
ACC	Adrenocortical carcinoma	77 / 128
BLCA	Bladder Urothelial Carcinoma	404 / 28
BRCA	Breast invasive carcinoma	1085 / 291
CESC	Cervical squamous cell carcinoma and endocervical adenocarcinoma	306 / 13
CHOL	Cholangio carcinoma	36 / 9
COAD	Colon adenocarcinoma	275 / 349
DLBC	Lymphoid Neoplasm Diffuse Large B-cell Lymphoma	47 / 337
ESCA	Esophageal carcinoma	182 / 286
GBM	Glioblastoma multiforme	163 / 207
HNSC	Head and Neck squamous cell carcinoma	519 / 44
KICH	Kidney Chromophobe	66 / 53
KIRC	Kidney renal clear cell carcinoma	523 / 100
KIRP	Kidney renal papillary cell carcinoma	286 / 60
LAML	Acute Myeloid Leukemia	173 / 70
LGG	Brain Lower Grade Glioma	518 / 207
LIHC	Liver hepatocellular carcinoma	369 / 160
LUAD	Lung adenocarcinoma	483 / 347
LUSC	Lung squamous cell carcinoma	486 / 338
OV	Ovarian serous cystadenocarcinoma	426 / 88
PAAD	Pancreatic adenocarcinoma	179 / 171
PCPG	Pheochromocytoma and Paraganglioma	182 / 3
PRAD	Prostate adenocarcinoma	492 / 152
READ	Rectal adenocarcinoma	92 / 318
SARC	Sarcoma	262 / 2
SKCM	Skin Cutaneous Melanoma	461 / 558
STAD	Stomach adenocarcinoma	408 / 211
TGCT	Testicular Germ Cell Tumors	137 / 165
THCA	Thyroid carcinoma	512/ 337
THYM	Thymoma	118 / 339
UCEC	Uterine Corpus Endometrial Carcinoma	174 / 91
UCS	Uterine Carcinosarcoma	57 / 78

Revised Supplementary Table 2. IC50 values with statistics in response to proteasome inhibitors

TAK-243	H1299					HCT116				HCT116				HCT116-p53KO			
	GFP		NRF3#1			GFP		NRF3		shCont		shNRF3		shCont		shNRF3	
	#1	#2	#1	#2	mtPOMP	#1	#2	#1	#2	#1	#2	#1	#2	#1	#2	#1	#2
IC50 (μM, Best-fit values)	0.79	0.52	0.61	0.79	0.68	1.53	1.37	1.45	1.80	1.13	1.02	1.25	1.42	1.81	2.04	1.59	1.62
IC50 (μM, Std. Error)	0.20	0.28	0.12	0.27	0.15	0.25	0.32	0.40	0.36	0.10	0.11	0.25	0.23	0.13	0.33	0.32	0.09
R square (Goodness of Fit)	0.91	0.91	0.92	0.97	0.92	0.94	0.91	0.93	0.94	0.93	0.93	0.85	0.94	0.99	0.98	0.95	1.00

BTZ	H1299					HCT116				HCT116				HCT116-p53KO			
	GFP		NRF3#1			GFP		NRF3		shCont		shNRF3		shCont		shNRF3	
	#1	#2	#1	#2	mtPOMP	#1	#2	#1	#2	#1	#2	#1	#2	#1	#2	#1	#2
IC50 (μM, Best-fit values)	11.39	15.01	34.13	49.49	12.28	13.82	13.19	52.61	53.28	17.48	24.34	12.49	19.58	72.52	49.63	17.65	16.82
IC50 (μM, Std. Error)	2.01	0.44	2.08	1.36	0.59	1.41	0.57	11.47	6.46	1.36	1.60	1.11	1.60	13.29	5.53	0.61	0.82
R square (Goodness of Fit)	0.99	0.99	0.98	0.99	0.97	0.98	0.98	0.95	0.97	0.97	0.98	0.97	0.99	1.00	0.98	0.99	0.98

Revised Supplementary Table 3. Primer and oligonucleotide sequences

qPCR primer

Target gene	Forward primer	Reverse primer
PSMA1 (α6)	CCAGGGCAGGATTCATCA	TCTGATTGCGCCCTTTTC
PSMA2 (α2)	GCCCCGATTACAGAGTGC	TGGACGAACACCACCTGA
PSMA3 (α7)	TGTTGATCGGCATGTTGG	TGGCCACTCTGTCTGCAA
PSMA4 (α3)	CATTGGCTGGGATAAGCA	ATGCATGTGGCCTTCCAT
PSMA5 (α5)	CTTCAGAGGGTGCCCAGA	CAGGCTGCACTGTGGCTA
PSMA6 (α1)	AACCAGGGTGGCCTTACA	CCGGTCATCACACAACCA
PSMA7 (α4)	AGTCAGTGCGCGAGTTCC	TGCCACCTGACTGAACCA
PSMB1 (β6)	CATGCTACAGCCCCTGCT	GCATGGCTCTGTCCAAGG
PSMB2 (β4)	CTTCACACGCCGAAACCT	AGGCTGCCAGGTAGTCCA
PSMB3 (β3)	TGGTGGCCAACCTCTTGT	GGCAGCCGATGAGGTCTA
PSMB4 (β7)	TCTCGGCCAGATGGTGAT	CACATAACCGAGGAAGCT
PSMB5 (β5)	CCATGGGCACCATGATCT	GAAGGTGGCCCCTGAAAT
PSMB6 (β1)	CTGATGGCGGGAATCATC	CCAATGGCAAAGGACTGC
PSMB7 (β2)	CGGCTGTGTCGGTGTATG	GCCAGTTTTCCGGACCTT
PSMC1 (Rpt2)	CATGGCCACAAACCGAAT	ATCAGGCAGGGGGAACTC
PSMC2 (Rpt1)	TGGGATTTGGCTGCAGAT	TTTGGGTCCTCCGAATCA
PSMC3 (Rpt5)	GCCCACGGAGCAATACAG	CATCAGCACCCCTTTTGG
PSMC4 (Rpt3)	GGAAGACCATGTTGGCAAAG	AAGATGATGGCAGGTGCATT
PSMC5 (Rpt6)	CTCCAGGCACTGGGAAGA	CGTGCCATGACAAACAGC
PSMC6 (Rpt4)	GGCAGATCGTGGGTGAAG	CGACGACAACCCACAACA
PSMD1 (Rpn2)	ATGTCAGGAGGGCAGCAG	AGCGCACATGAGGGTTGT
PSMD2 (Rpn1)	CCCAAGGTGCCTGATGAC	CTTGGCCAAAAGCTGCAT
PSMD3 (Rpn3)	GCTGTGCAGGGCTTCTTC	GGTGTCGACGCAGCTTTT
PSMD4 (Rpn10)	CGGGATTGCTACGACTGG	CAGTGCGGCCAAACTCTT
PSMD6 (Rpn7)	GGCAAAGGCCGAGTACCT	ACCCAGGGCCACAGTTTT
PSMD7 (Rpn8)	GCTGGCAGTGCAGAAGGT	ACCCCAAAAGCACACCAA
PSMD8 (Rpn12)	CCACCCGGATCCTCTTCT	GCTGGCTGGCAAAACTGT
PSMD11 (Rpn6)	ATGCAGGGAGGCAGACAG	GGAGCTCTGCCCGGTAAT
PSMD12 (Rpn5)	TCCAAAAGGCGGAGTCAG	GCCTTCGGTAACCATTCG
PSMD13 (Rpn9)	ATGCTCTGCGGTTTTTGG	CAGCACAGGGTGCATGAG
PSMD14 (Rpn11)	CCGTGCTGGAGTTCCAAT	TGCCTCCACACTGACACC
ADRM1 (Rpn13)	CCCCTCATGTGCCAGTTC	TTCGTGTCGCCCTCTTTC
SHFM1 (Rpn15)	GAAAAAGCAGCCGGTAGA	AAGCCAGCCCAGTCTTCG
PSME1 (PA28α)	CATCCCAGTGCCTGATCC	GCCACAGGGAGGACCTTT
PSME2 (PA28β)	CGCAAACAGGTGGAGGTC	CCCGGAGGGAAGTCAAGT
PSME3 (PA28γ)	CAGCCTTCGGCTCATCAT	GGGGCCGTTTGATCTTCT
PSME4 (PA200)	GAAAGCACCCAGCGATGT	GGCACAGAAGCTCCCAAA
PSMG1(PAC1)	CCAATCCCTCGGTTTTTC	CCTTGGACAAGAGCCAAA
PSMG2 (PAC2)	CCGGGAGGAGGTATCACA	TGGGATGTTGTCCCCTTC
PSMG3 (PAC3)	GTCCTTCTGGGGCAGGAT	TCACCACACCTGGCACAC
PSMG4 (PAC4)	CGGACTCGCTGTTCCTGT	TGGGCAAGGCCAGTAGAG
POMP (UMP1)	AGGCAGTGCAGCAGGTTC	GGCTCTCCCATGACTTCG
NRF3	CTGACTGGGAGGCAGAAAAG	TCAGGCTGTGATGAAAGCAA
p53	GCCCAACAACACCAGCTCCT	CCTGGGCATCCTTGAGTTCC
Retinoblastoma	AGGCCCCCTACCTTGTCA	TGTTGGTGTTGGCAGAC
p21	GGAGACTCTCAGGGTCGAAA	TTAGGGCTTCCTCTTGGAGA
PUMA	GGGCCCAGACTGTGAATCCT	ACGTGCTCTCTCTAAACCTATGCA
β-actin	CCAACCGCGAGAAGAT	CCAGAGGCGTACAGGG
HPRT	TTCCTTGGTCAGGCAGTATAATCC	AGTCTGGCTTATATCCAACACTTCG

Revised Supplementary Table 3. ***Continued***

ChIP primer

**Traget region**	**Forward primer**	**Reverse primer**
p21-p53RE	GTGGCTCTGATTGGCTTTCTG	CTGAAAACAGGCAGCCCAAG
PUMA-p53RE	GCGAGACTGTGGCCTTGTGT	CGTTCCAGGGTCCACAAAGT
POMP-ARE	CCTCGGAAACGGAAGTGA	ACCATCTTCCGCAGCTCT
POMP-negative locus	GGGCTTTTTGGCCTCTGT	TGGTTGCCCACAAGTCCT

siRNA

**Target gene**	**Sense**	**Antisense**
siControl	UUCUCCGAACGUGUCACGUTT	ACGUGACACGUUCGGAAATT
siNRF3 #1	GGAUCAAAGUGAUUCUGAUTT	AUCAGAAUCACUUUGAUCCAA
siNRF3 #2	GCAAAGAAGGAAACUCUUATT	UAAGAGUUUCCUUCUUUGCUU

shRNA*

**Target gene**	**Upper**	**Lower**
shControl	TCGAGGTTCTCCGAACGTGTCACGTT<br>TCAAGAGAACGTGACACGTTCGGAGA<br>ATTTTTTACGCGTA	AGCTTACGCGTAAAAAATTCTCCGAA<br>CGTGTCACGTTCTCTTGAAACGTGAC<br>ACGTTCGGAGAACC
shNRF3 #1	TCGAGGGGATCAAAGTGATTCTGATTT<br>CAAGAGAATCAGAATCACTTTGATCC<br>TTTTTTACGCGTA	AGCTTACGCGTAAAAAAGGATCAAAG<br>TGATTCTGATTCTCTTGAAATCAGAAT<br>CACTTTGATCCCC
shNRF3 #2	TCGAGGGCAAAGAAGGAAACTCTTAT<br>TCAAGAGATAAGAGTTTCCTTCTTTGC<br>TTTTTTACGCGTA	AGCTTACGCGTAAAAAAGCAAAGAAG<br>GAAACTCTTATCTCTTGAATAAGAGTT<br>TCCTTCTTTGCCC

***These shRNAs have the identical target sequences with siRNAs.**

guide RNA

**Name**	**Upper**	**Lower**
POMP-ARE-gRNA	CACCGTCGGAAACGGAAGTGAGCGG	AAACCCGCTCACTTCCGTTTCCGAC

DNA sequence primer

**Name**	**Forward**	Reverse
ChIP region#1 (non-ARE)	TTCTCCTGCTCCCAACAACT	CAGCCTAGGTGACACAGCAA
ChIP region#2 (*POMP*-ARE)	CCTCGGAAACGGAAGTGA	ACCATCTTCCGCAGCTCT
